# Variation of Neonatal Outcomes and Care Practices for Preterm Infants <34 Weeks' Gestation in Different Regions of China: A Cohort Study

**DOI:** 10.3389/fped.2021.760646

**Published:** 2021-11-11

**Authors:** Ruimiao Bai, Siyuan Jiang, Jinzhen Guo, Shanyu Jiang, Shoo K. Lee, Zhankui Li, Yun Cao

**Affiliations:** ^1^Department of Neonatology, Northwest Women's and Children's Hospital, Xi'an, China; ^2^Department of Neonatology, Children's Hospital of Fudan University, Shanghai, China; ^3^NHC Key Laboratory of Neonatal Diseases (Fudan University), Children's Hospital of Fudan University, Shanghai, China; ^4^Department of Neonatology, The Affiliated Wuxi Maternity and Child Health Hospital of Nanjing Medical University, Nanjing, China; ^5^Maternal-Infant Care Research Centre and Department of Pediatrics, Mount Sinai Hospital, Toronto, ON, Canada; ^6^Department of Pediatrics, University of Toronto, Toronto, ON, Canada; ^7^Department of Obstetrics and Gynecology, Dalla Lana School of Public Health, University of Toronto, Toronto, ON, Canada

**Keywords:** preterm infants, discharge against medical advice, intraventricular hemorrhage, periventricular leukomalacia, bronchopulmonary dysplasia, necrotizing enterocolitis, sepsis, China

## Abstract

**Background:** To compare outcomes and care practices of preterm infants born at <34 weeks' gestation in the different regions of China from 2015 to 2018.

**Methods:** This cohort study enrolled all infants born at <34 weeks and admitted to 25 tertiary neonatal intensive care units across China from May 1st, 2015, to April 30th, 2018. The participating hospitals were categorized into three groups according to their distinct geographic locations: eastern China, central China, and western China. Multilevel mixed-effects logistic regression models were used to assess the independent association between neonatal outcomes and regions.

**Results:** A total of 27,532 infants at <34 weeks' gestation were enrolled in our study. Overall, 14,178 (51.5%) infants were from 12 hospitals in eastern China, 8,069 (29.3%) from 9 hospitals in central China, and 5,285 (19.2%) from 4 hospitals in western China. Infants in eastern China had the lowest rates of mortality or any morbidity (23.3%), overall mortality (7.6%), in-hospital mortality (3.7%), and discharge against medical advice (DAMA, 6.3%), compared with central (27.8, 11.3, 5.0, and 10.6%, respectively) and western China (37.4, 19.4, 7.7, and 19.4%, respectively). Multilevel mixed-effects logistic regression showed that infants in western China were exposed to the highest risks of mortality or any morbidity, overall mortality, in-hospital mortality, and DAMA. Significant variations of care practices existed in three regions. Infants in central China had the longest duration of the first course of invasive ventilation, the lowest rate of continuous positive airway pressure within 24 h after birth, the lowest rate of breast milk feeding, the latest initiation of feeds, and the longest duration of total parenteral nutrition among the three regions.

**Conclusions:** We identified marked disparities in outcomes and clinical care practices of preterm infants born at <34 weeks' gestation in different regions of China. Targeted quality improvement efforts are needed to improve the outcomes of premature infants in different regions of China.

## Introduction

There has been impressive progress in maternal and child health in China, with a fast reduction in under-5 mortality during the past two decades (1–3). However, persistent regional disparities in maternal, neonatal, and child health have also been recognized (1, 2). These identified disparities have provided valuable information to inform region-specific strategies for further improvements in maternal and child outcomes in different regions (1). Preterm birth is the leading cause of mortality and morbidity among children younger than 5 years (4, 5), accounting for 75% of perinatal deaths and more than half of perinatal morbidity (6–8). Preterm infants born at <34 weeks' gestation are the highest risks of mortality and morbidities (9). Although currently no nation-level study has provided data on the variation of outcomes and healthcare practices for preterm infants in different regions of China.

The Chinese Government divided China into three regions, namely, eastern, central, and western China, based on disparities in economic progress. This division is still to be used today for the allocation of resources from the central government (1). The eastern region is the most urbanized area with the highest gross domestic product (GDP) per capita in China, whereas the western region is the most rural area with the lowest GDP per capita. In this study, we used the largest cohort of preterm infants at <34 weeks' gestation to explore the variations of neonatal outcomes and care practices of premature infants in the three regions of China. We hope that these data will contribute to identifying region-specific problems existing in the healthcare of preterm infants and to informing targeted regional strategies and quality improvement initiatives for improvements in neonatal outcomes in different regions.

## Materials and Methods

### Study Design and Patient Population

This cohort study included all infants at <34 weeks' gestation who were admitted to 25 tertiary neonatal intensive care units (NICUs) across China from May 1, 2015, to April 30, 2018, within 7 days after birth. Stillborn and delivery room deaths were not eligible. Infants were followed until death or discharge from the NICU. The ethics committee of the Children's Hospital of Fudan University approved the study [no. 28 (2015)].

Clinical data of all eligible infants were prospectively collected using a standardized clinical database. The clinical database was initially established for a cluster randomized controlled trial “Reduction of Infection in Neonatal Intensive Care Units using the Evidence-based Practice for Improving Quality (EPIQ)” (REIN-EPIQ study, clinicaltrials.gov, #NCT02600195). All data collection followed a standard manual of operations and definitions (10).

### Sites and Regional Categories

**Figure 1:**
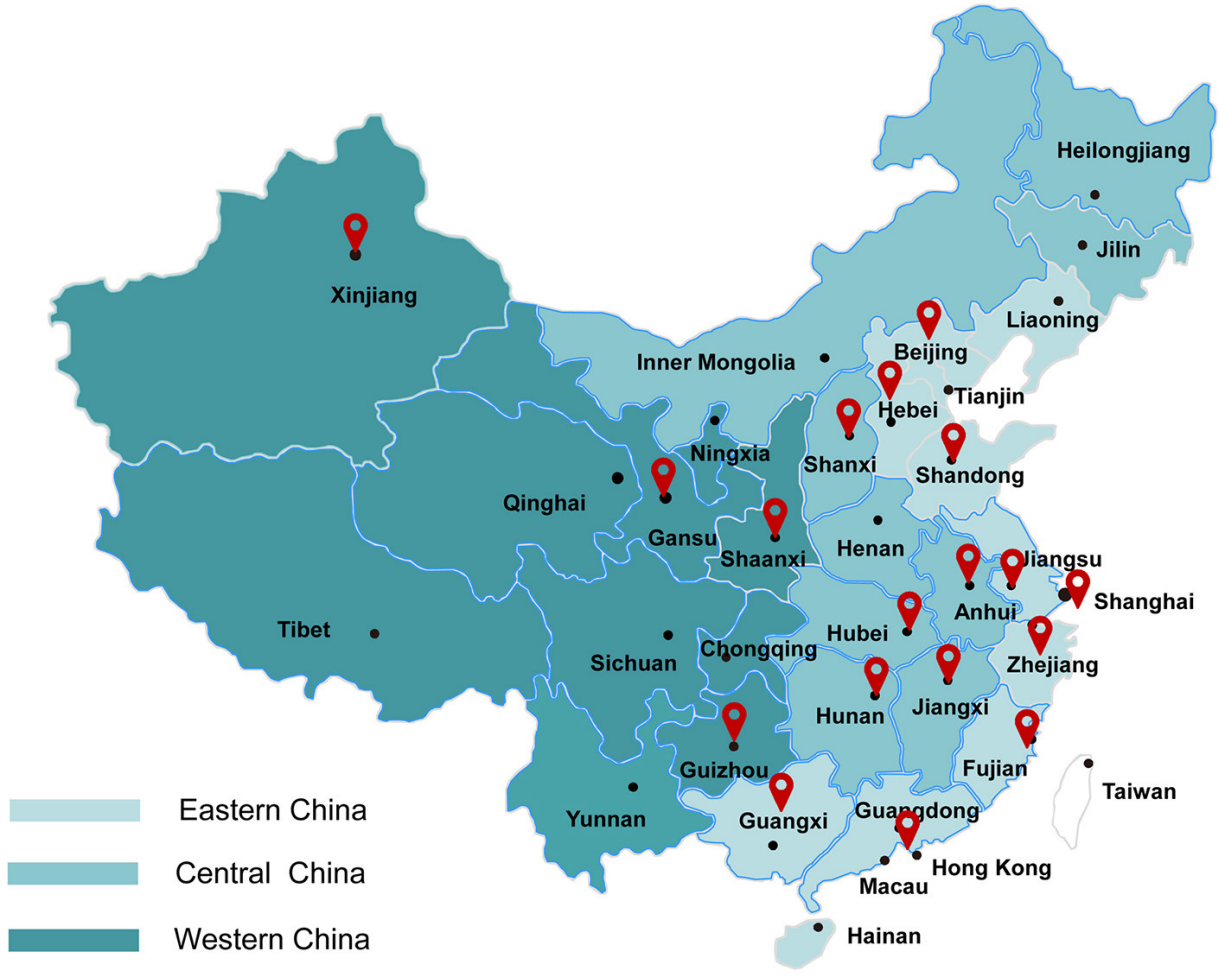
Geographical distribution of eastern, central, and western regions in China and locations of the participating hospitals (1, 2). Eastern China: Shanghai First Maternity and Infant Hospital; Qingdao Women and Children's Hospital; Women's Hospital of Nanjing Medical University; Qilu Children's Hospital of Shandong University; The Affiliated Wuxi Maternity and Child Health Care; Children's Hospital of Nanjing Medical University; Fujian Provincial Maternity and Children's Hospital; The 2nd Affiliated Hospital and Yuying Children's Hospital of Wenzhou Medical University; Beijing Children's Hospital of Capital Medical University; The Affiliated Shenzhen Maternity and Child Healthcare of Southern Medical University; Suzhou Municipal Hospital; Children's Hospital of Fudan University. Central China: The Maternal and Child Health Hospital of Guangxi Zhuang Autonomous Region; Women and Children's Hospital of Hubei Province; Tongji Hospital, Tongji Medical College, Huazhong University of Science and Technology; The Third Xiangya Hospital of Central South University; Children's Hospital Affiliated to Zhenzhou University; The First Affiliated Hospital of Anhui Medical University; Jiangxi Provincial Children's Hospital; Children's Hospital of Shanxi/Women Health Center of Shanxi; Children's Hospital of Hebei Province. Western China: Northwest Women and Children's Hospital; Gansu Provincial Maternity and Child-care Hospital; First Affiliated Hospital of Xinjiang Medical University; Guiyang Maternal and Child Health Care Hospital.

A total of 25 hospitals from 19 provinces participated in the study. These participating hospitals were categorized according to their geographic locations into three groups, which were eastern China, central China, and western China (Figure 1) (1, 2). There were 12 hospitals from eastern China, 9 hospitals from central China, and 4 hospitals from western China. All hospitals have facilities to provide comprehensive care for infants with gestational age at <28 weeks or birth weight <1,000 g. Seventeen hospitals were national or provincial neonatal referral centers, and eight were regional referral centers in metropolitan cities. The characteristics of three groups of hospitals are shown in Supplementary Table 1. Overall, 5 of 12 (41.7%), 3 of 9 (33.3%), and 1 of 4 (25%) hospitals were freestanding children's hospitals in eastern, central, and western China, respectively. All hospitals included from the eastern regions were teaching hospitals. A total of 5 of 9 (55.6%) and 2 of 4 (50%) included hospitals in central and western China were teaching hospitals, respectively. Overall, 66.7% of hospitals had dedicated neonatal transport teams in eastern and central China, 3 of 4 (75%) hospitals had dedicated neonatal transport teams in western China. All hospitals in central and western China and 7 of 12 (58.3%) hospitals in eastern China had the capacity to perform neonatal general surgery. All hospitals included from the central regions were capable of performing PDA ligation. A total of 8 of 12 (66.7%) and 3 of 4 (75%) hospitals in eastern and western China were capable of performing PDA ligation, respectively.

### Definitions

Mortality or any morbidity was defined as overall death or any of the major morbidities, including sepsis, necrotizing enterocolitis (NEC), severe intraventricular hemorrhage (IVH), periventricular leukomalacia (PVL), severe retinopathy of prematurity (ROP), and bronchopulmonary dysplasia (BPD). Discharge against medical advice (DAMA) was defined as a situation where parents terminated treatment before the treating physicians recommended discharge according to discharge criteria in participating NICUs. DAMA infants were not followed after NICU discharge. Therefore, we used predefined criteria to predict the likelihood of death for DAMA infants (10). If infants required invasive or noninvasive mechanical ventilation, inotropes infusion, or total parenteral nutrition (TPN; no enteral feeds initiated) on the day of discharge, we considered that they would not survive after discharge. In-hospital mortality was defined as death in NICUs among infants who received complete care. Overall mortality was defined as the combination of in-hospital deaths for infants with complete care and deaths after discharge for DAMA infants. Sepsis was defined as infections with positive blood or cerebrospinal fluid cultures (11). NEC was defined as stage ≥2, according to Bell's criteria (12, 13). Severe IVH was defined as grade ≥ 3 IVH according to Papile et al. (14). PVL was defined as the presence of periventricular cysts on cranial ultrasound or cranial magnetic resonance imaging (MRI) scans. Severe ROP was defined as stage ≥3 according to the International Classification of ROP (15). There has been national guideline on ROP screening protocol that all hospitals should follow. Although there is no unified guideline for IVH screening in China, the majority of hospitals would perform the first head ultrasound within 7 days for very preterm infants. There is no universal MRI scan indication in preterm infants in China; however, the majority of hospitals perform predischarge or full-term MRI scan for all preterm infants survived. We did not standardize the diagnostic protocol for both morbidities in our study, which might result in some misclassifications. BPD was defined as mechanical ventilation or needed for supplemental oxygen at 36 weeks' postmenstrual age or discharge (16).

Gestational age was calculated from the best obstetric estimate based on early prenatal ultrasonography, last menstrual period, obstetric examination, or all three. Small for gestational age (SGA) was defined as birth weight <10th percentile for the gestational age according to the Chinese neonatal birth weight values (17). Transport Risk Index of Physiologic Stability (TRIPS) score was used as an illness severity score on NICU admission, which consisted of four physiologic variables: temperature, blood pressure, respiratory status, and response to noxious stimuli upon initial admission to the NICU (18, 19).

### Statistical Analyses

Descriptive statistical methods were used to describe the study population. Maternal and neonatal characteristics were compared using the Kruskal–Wallis test for continuous variables and χ^2^ tests for categorical variables among three groups of different regions. Group pairwise comparisons were further conducted where the omnibus test was significant. For the purpose of multiple comparison adjustment, *p* = 0.017 was considered significant according to the Bonferroni method.

Multilevel mixed-effects logistic regression models were used to assess the independent association between neonatal outcomes and regions, accounting for the intracluster correlation among infants within individual sites. Sites were considered as independent clusters with random effects in the models. Two kinds of models were generated with different patient-level variates adjusted. In model 1, the patient-level covariates included gender, gestational age, SGA, maternal hypertension, and maternal diabetes, which were the most important determinants of neonatal outcomes and were not influenced by different care practices. In model 2, perinatal practices, including inborn, prenatal care, antenatal steroids, primigravida, and cesarean section, were further adjusted. Repeat analyses were done in subgroups of infants with gestational age at <28 weeks, 28–31 weeks, and 32–33 weeks.

A two-sided *p* < 0.05 was used to determine statistical significance. Statistical analysis was performed using Stata 15.0 (StataCorp, College Station, TX, USA).

## Results

### Neonatal and Maternal Characteristics

A total of 27,532 infants with gestational age at <34 weeks were admitted to participating NICUs during the study period and were enrolled in our study. Overall, 14,178 (51.5%) infants were from 12 hospitals in eastern China, 8,069 (29.3%) from 9 hospitals in central China, and 5,285 (19.2%) from 4 hospitals in western China. The infant and maternal characteristics of infants are presented in Table 1. There were higher proportions of infants with the lowest gestational ages (≤ 27^+6^ weeks) or birth weight (≤ 1,249 g) in eastern China, compared with hospitals in central and western China. Infants in eastern China were least likely to be SGA and had the lowest TRIPS score on admission. Mothers in eastern China were most likely to receive prenatal care and antenatal steroids, compared with mothers in central and western China. Infants admitted to NICUs in central China showed the lowest Apgar scores and highest TRIPS score on admission and were least likely to be inborn, compared with infants in eastern and western China. Mothers in western China had the lowest rate of prenatal care and antenatal steroids in all three regions.

**Table 1:** Characteristics of the infants and maternal admitted to NICUs in three regions of China during 2015–2018.

	**Eastern China**	**Central China**	**Western China**	***p***
	***n* = 14,178**	***n* = 8,069**	***n* = 5,285**	
**Infant characteristics**				
GA, median (IQR)	31.2 (29.9–32.9)^2, 3^	31.4 (30.1–33.0)^1, 3^	31.4 (30.1–32.9)^1, 2^	<0.001
<26 weeks, *n* (%)	240 (1.7)	57 (0.7)	33 (0.6)	
26–27 weeks, *n* (%)	948 (6.7)	362 (4.5)	230 (4.4)	
28–31 weeks, *n* (%)	6,593 (46.5)	3,725 (46.2)	2,489 (47.1)	
32–33 weeks, *n* (%)	6,397 (45.1)	3,925 (48.6)	2,533 (48.0)	
BW, mean (SD)	1,602 ± 426^2^	1,642 ± 410^1, 3^	1,616 ± 390^2^	<0.001
<750 g, *n* (%)	186 (1.3)	38 (0.5)	27 (0.5)	
750–999 g, *n* (%)	855 (6.0)	345 (4.3)	201 (3.8)	
1,000–1,249 g, *n* (%)	2,008 (14.2)	1,018 (12.6)	675 (12.8)	
1,250–1,499 g, *n* (%)	2,792 (19.7)	1,610 (20.0)	1,093 (20.7)	
1,500–1,999 g, *n* (%)	5,644 (39.8)	3,321 (41.2)	2,399 (45.4)	
≥2,000 g, *n* (%)	2,693 (19.0)	1,736 (21.5)	890 (16.8)	
Male, *n* (%)	8,017 (56.6)^2^	4,773 (59.2)^1, 3^	3,011 (57.0)^2^	<0.001
SGA, *n* (%)	1,666 (11.8)^2, 3^	1,173 (14.5)^1^	789 (14.9)^1^	<0.001
1-min Apgar ≤ 3, *n* (%)	611 (4.4)^2^	481 (6.5)^1^	262 (5.1)^2^	<0.001
5-min Apgar ≤ 3, *n* (%)	142 (1.1)^2^	113 (1.7)^1, 3^	51 (1.0)^2^	<0.001
TRIPS score, median (IQR)	8 (6–13)^2, 3^	14 (8–22)^1, 3^	12 (6–21)^1, 2^	<0.001
Major congenital anomaly	241 (1.7)	129 (1.6)	82 (1.6%)	0.720
**Maternal characteristics**				
Maternal hypertension, *n* (%)	2,158 (15.3)^2, 3^	1,390 (17.6)^1, 3^	1,031 (19.8)^1, 2^	<0.001
Maternal diabetes, *n* (%)	2,224 (15.8)^2, 3^	677 (8.6)^1, 3^	338 (6.5)^1, 2^	<0.001
**Perinatal practices**				
Inborn, *n* (%)	9,829 (69.3)^2, 3^	4,942 (61.3)^1, 3^	4,408 (83.4)^1, 2^	<0.001
Prenatal care, *n* (%)	14,038 (99.2)^2, 3^	7,761 (98.5)^1, 3^	5,085 (97)^1, 2^	<0.001
Antenatal steroids, *n* (%)	9,705 (71.6)^2, 3^	4,449 (60.1)^1^	3,045 (58.8)^1^	<0.001
Primigravida, *n* (%)	4,997 (35.3)^2^	3,018 (37.4)^1, 3^	1,804 (34.1)^1, 2^	<0.001
Cesarean section, *n* (%)	7,847 (55.4)^2^	4,243 (52.6)^1^	2,836 (53.7)	<0.001

1, 2, 3 *The superscripts indicate the significant pairwise comparison, of which 1, 2, and 3 correspond to comparing with eastern, central, and western China, respectively*.

*IQR, interquartile range*.

### Neonatal Outcomes in Different Regions of China

Neonatal outcomes of preterm infants in the three regions are shown in Table 2 and Supplementary Table 2. Infants admitted to NICUs in eastern China had the lowest rates of mortality or any morbidity (23.3%), overall mortality (7.6%), and in-hospital mortality (3.7%), compared with the other two regions. Infants from western China showed the highest rates of mortality or any morbidity (37.4%), overall mortality (19.4%), and in-hospital mortality (7.7%). Although with the lowest DAMA rate among three regions, there remained 6.3% of infants in eastern China discharged against medical advice, and DAMA rates in central and western China were as high as 11.3 and 19.4%, respectively. Subgroup analysis in different gestational age groups showed similar results (Supplementary Table 2), except for infants ≤ 27^+6^ weeks. For infants ≤ 27^+6^ weeks, although eastern China also showed the lowest rates of mortality, rates of mortality or any morbidity were not significantly different among the three regions.

**Table 2:** Comparison of unadjusted outcomes for preterm infants admitted to NICUs in three regions of China.

	**Eastern China**	**Central China**	**Western China**	***p***
	***n* = 14,178**	***n* = 8,069**	***n* = 5,285**	
Mortality or any morbidity,^*^n/*N* (%)	3,304/14,178 (23.3)^2, 3^	2,242/8,069 (27.8)^1, 3^	1,977/5,285 (37.4)^1, 2^	<0.001
**Mortality**				
Overall mortality, *n*/*N* (%)	1,078/14,178 (7.6)^2, 3^	909/8,069 (11.3)^1, 3^	1,023/5,285 (19.4)^1, 2^	<0.001
In-hospital mortality, *n*/*N* (%)	492/13,287 (3.7)^2, 3^	362/7,215 (5.0)^1, 3^	334/4,348 (7.7)^1, 2^	<0.001
DAMA, *n*/*N* (%)	891/14,178 (6.3)^2, 3^	854/8,069 (10.6)^1, 3^	937/5,285 (17.7)^1, 2^	<0.001
**Morbidities**				
Sepsis, *n*/*N* (%)	761/14,178 (5.4)^2, 3^	313/8,069 (3.9)^1, 3^	363/5,285 (6.9)^1, 2^	<0.001
Severe IVH or PVL, *n*/*N* (%)	717/12,801 (5.6)^2^	696/7,100 (9.8)^1, 3^	260/4,591 (5.7)^2^	<0.001
NEC, *n*/*N* (%)	542/13,479 (4.0)^2, 3^	191/7,617 (2.5)^1, 3^	227/4,629 (4.9)^1, 2^	<0.001
BPD, *n*/*N* (%)	1,872/14,178 (13.2)^3^	1,201/8,069 (14.9)^3^	1,396/5,285 (26.4)^1, 2^	<0.001
Severe ROP, *n*/*N* (%)	127/7,422 (1.7)^3^	72/4,093 (1.8)^3^	18/2,493 (0.7)^1, 2^	<0.001

1, 2, 3 *The superscripts indicate the significant pairwise comparison of which 1, 2 and 3 correspond to comparing with eastern, central and western China, respectively*.

* *Mortality or any morbidity was defined as the occurrence of overall death or any morbidity, including sepsis; NEC, necrotizing enterocolitis; IVH, severe intraventricular hemorrhage; PVL, periventricular leukomalacia; ROP, severe retinopathy of prematurity; BPD, bronchopulmonary dysplasia*.

For major morbidities, infants in western China showed the highest rates of sepsis, NEC, and BPD, but the lowest incidence of severe ROP (Table 2; Supplementary Table 2). Infants from central China showed the lowest incidences of sepsis and NEC, but had the highest incidence of severe IVH or PVL.

After adjustment for infant and maternal baseline characteristics, infants in eastern China were at the lowest risks of overall mortality, in-hospital mortality, and DAMA (Table 3). Infants in western China were generally exposed to the highest risks of mortality or any morbidity, overall mortality, in-hospital mortality, and DAMA (Table 3). The association of neonatal outcomes and regions remained similar after additional adjustment of perinatal care practices (Table 3). Subgroup analyses by different gestational ages are shown in Supplementary Table 3. For infants ≤ 27^+6^ weeks, risks of mortality or any morbidity and DAMA were not significantly different among the three regions.

**Table 3:** Comparison of adjusted outcomes for preterm infants admitted to NICUs in three regions of China.

		**Model 1**		**Model 2**	
	**Eastern China**	**Central China**	**Western China**	**Central China**	**Western China**
Mortality or any morbidity	Reference	1.4 (0.9–2.4)	2.6 (1.3–5.2)	1.4 (0.8–2.3)	2.8 (1.4–5.3)
Overall mortality	Reference	1.8 (1.1–2.9)	3.9 (2.1–7.4)	1.6 (1.0–2.5)	4.1 (2.2–7.3)
In-hospital mortality	Reference	1.6 (1.0–2.6)	3.6 (2.0–6.5)	1.5 (0.9–2.3)	3.7 (2.1–6.5)
DAMA	Reference	1.9 (1.1–3.1)	3.3 (1.7–6.3)	1.7 (1.1–2.8)	3.3 (1.8–6.2)
Sepsis	Reference	0.8 (0.4–1.4)	1.4 (0.6–3.1)	0.8 (0.4–1.4)	1.4 (0.7–3.2)
Severe IVH or PVL	Reference	2.2 (1.1–4.7)	1.7 (0.7–4.4)	2.3 (1.1–5.0)	1.9 (0.8–5.0)
NEC	Reference	0.6 (0.3–1.2)	0.9 (0.4–2.1)	0.6 (0.3–1.2)	0.9 (0.4–2.2)
BPD	Reference	1.1 (0.5–2.3)	2.2 (0.8–5.5)	1.1 (0.5–2.2)	2.2 (0.9–5.6)
Severe ROP	Reference	1.5 (0.7–3.1)	0.7 (0.3–1.8)	1.5 (0.7–3.2)	0.7 (0.3–2.0)

*Multilevel mixed-effects logistic regression models were used, accounting for the intracluster correlation among infants within individual sites*.

*Patient-level covariants adjusted in models*.

*Model 1: gender, gestational age, SGA, maternal hypertension, maternal diabetes*.

*Model 2: gender, gestational age, SGA, maternal hypertension, maternal diabetes, inborn, prenatal care, antenatal steroids, primigravida, cesarean section*.

### NICU Care Practices in Different Regions of China

Significant variations of care practices existed in three regions (Table 4). For respiratory support, infants in eastern China showed the lowest rates of invasive ventilation within 24 h and during hospitalization, compared to central and western China. Infants in central China had the longest duration of the first course of invasive ventilation and were least likely to receive continuous positive airway pressure (CPAP) within 24 h after birth. For nutrition practices, central China showed the lowest rate of breast milk feeding, latest initiation of feeds, and the longest duration of TPN among three regions. Among infants born at 32^+0^+33^+6^weeks, the rate of invasive ventilation and CPAP within 24 h was highest in western China, together with the longest duration of TPN and NICU stays.

**Table 4:** Care practices for preterm infants admitted to NICUs in different regions of China.

	**Gestation age**	**Eastern China**	**Central China**	**Western China**	***p***
**Ventilation practices**					
Use of CPAP within 24 h, *n*/*N* (%)	≤ 27^+6^	679/1,188 (57.2%)^2, 3^	141/419 (33.7%)^1, 3^	124/263 (47.1%)^1, 2^	<0.01
	28^+0^-31^+6^	4,046/6,593 (61.4%)^2^	1,885/3,725 (50.6%)^1, 3^	1,479/2,489 (59.4%)^2^	<0.01
	32^+0^-33^+6^	2,422/6,397 (37.9%)^2, 3^	1,290/3,925 (32.9%)^1, 3^	1,125/2,533 (44.4%)^1, 2^	<0.01
	Total	7,147/14,178 (50.4%)^2^	3,316/8,069 (41.1%)^1, 3^	2,728/5,285 (51.6%)^2^	<0.01
Invasive ventilation within 24 h, *n*/*N* (%)	≤ 27^+6^	709/1,188 (59.7%)	263/419 (62.8%)	143/263 (54.4%)	0.09
	28^+0^-31^+6^	1,560/6,593 (23.7%)^2, 3^	1,072/3,725 (28.8%)^1^	686/2,489 (27.6%)^1^	<0.01
	32^+0^-33^+6^	670/6,397 (10.5%)^2, 3^	610/3,925 (15.5%)^1^	442/2,533 (17.4%)^1^	<0.01
	Total	2,939/14,178 (20.7%)^2, 3^	1,945/8,069 (24.1%)^1^	1,271/5,285 (24.0%)^1^	<0.01
Duration of first course of invasive ventilation (days) (IQR)^a^	≤ 27^+6^	2 (2, 6)^2, 3^	6 (3, 12)^1^	5 (3, 11)^1^	<0.01
	28^+0^-31^+6^	3 (2, 5)^2^	4 (3, 7)^1, 3^	3 (2, 5)^2^	<0.01
	32^+0^-33^+6^	3 (2, 5)^2^	4 (3, 6)^1, 3^	3 (2, 5)^2^	<0.01
	Total	3 (2, 5)^3^	4 (3, 7)^3^	3 (2, 5)^1, 2^	<0.01
Invasive ventilation during hospitalization, *n*/*N* (%)	≤ 27^+6^	884 /1,188 (74.4%)	313 /419 (74.7%)	182/263 (69.2%)	0.19
	28^+0^-31^+6^	2,082 /6,593 (31.6%)^2, 3^	1,372/3,725 (36.8%)^1, 3^	1,002/2,489 (40.3%)^1, 2^	<0.01
	32^+0^-33^+6^	856/6,397 (13.4%)^2, 3^	777/3,925 (19.8%)^1, 3^	567/2,533 (22.4%)^1, 2^	<0.01
	Total	3,822/14,178 (27%)^2, 3^	2,462/8,069 (30.5%)^1, 3^	1,751/5,285 (33.1%)^1, 2^	<0.01
**Nutrition practices**					
Breast milk feeding, n /N (%)^b^	≤ 27^+6^	772/1,188 (65.0%)^2, 3^	185/419 (44.2%)^1, 3^	140/263 (53.2%)^1, 2^	<0.01
	28^+0^-31^+6^	4,088/6,593 (62.0%)^2^	1,549/3,725 (41.6%)^1, 3^	1,601/2,489 (64.3%)^2^	<0.01
	32^+0^-33^+6^	3,349/6,397 (52.4%)^2, 3^	1,245/3,925 (31.7%)^1, 3^	1,505/2,533 (59.4%)^1, 2^	<0.01
	Total	8,209/14,178 (57.9%)^2, 3^	2,979/8,069 (36.9%)^1, 3^	3,246/5,285 (61.4%)^1, 2^	<0.01
Day of feeds initiation, median (IQR)	≤ 27^+6^	3 (2, 3)^2, 3^	3 (2, 5)^1, 3^	2 (1, 3)^1, 2^	<0.01
	28^+0^-31^+6^	2 (2, 3)^2, 3^	3 (2, 4)^1, 3^	2 (1, 2)^1, 2^	<0.01
	32^+0^-33^+6^	2 (1, 2)^2, 3^	2 (2, 4)^1, 3^	2 (1, 2)^1, 2^	<0.01
	Total	2 (2, 3)^2, 3^	3 (2, 4)^1, 3^	2 (1, 2)^1, 2^	<0.01
Duration of TPN (days), median (IQR)	≤ 27^+6^	36 (23, 50)	41 (24.5, 54)	34 (25, 44)	0.26
	28^+0^-31^+6^	19 (12, 29)^2^	22 (13, 32)^1, 3^	20 (13, 30)^2^	<0.01
	32^+0^-33^+6^	10 (6, 15)^2, 3^	11 (7, 17)^1, 3^	12 (8, 18)^1, 2^	<0.01
	Total	14 (8, 23)^2, 3^	15 (9, 26)^1^	15 (10, 24)^1^	<0.01
NICU stay(days), median (IQR) ^c^	≤ 27^+6^	76 (64, 92)^2, 3^	66.5 (57, 78)^1^	69 (58, 84)^1^	<0.01
	28^+0^-31^+6^	40 (29, 51)^2, 3^	37 (27, 48)^1, 3^	37 (28, 50)^1, 2^	<0.01
	32^+0^-33^+6^	18 (13, 26)^3^	18 (13, 26)^3^	21 (14, 30)^1, 2^	<0.01
	Total	28 (17, 44)^2^	27 (17, 40)^1, 3^	29 (18, 41)^2^	<0.01
PMA at discharge (weeks), median (IQR)^d^	≤ 27+6	37.7 (36.3,39.7)^2^	36.7 (35.5,38.1)^1^	37.1 (35.7,39.0)	<0.01
	28^+0^-31^+6^	35.9 (34.7,37.1)^2^	35.6 (34.6,37.0)^1, 3^	37.0 (28.0,50.1)^2^	<0.01
	32^+0^-33^+6^	35.6 (34.9,36.4)^3^	35.6 (34.9,36.6)^3^	35.7 (34.9,37.0)^1, 2^	<0.01
	Total	35.7 (34.9,36.9)^2^	35.6 (34.7,36.7)^1, 3^	35.9 (34.9,37.1)^2^	<0.01

1, 2, 3 *The superscripts indicate the significant pairwise comparison of which 1, 2, and 3 correspond to comparing with eastern, central, and western China, respectively*.

a *IQR, interquartile range*.

b *Any amount of breast milk*.

c *Excluding infants who died in the NICU*.

d *PMA, postmenstrual age*.

## Discussion

In this large, multicenter, prospective cohort study involving infants with gestational age at <34 weeks, we demonstrated marked disparities in mortality, morbidities, and care practices in different regions of China. This information will be valuable to guide region-specific efforts to improve the care and outcomes of preterm infants more efficiently.

We found that preterm infants admitted to NICUs in eastern China were at the lowest risks of death, whereas infants in western China had the highest mortality. Several studies have reported a similar disparity of maternal, child, and neonatal mortality from eastern to western China (1–3, 20–23). Overall, inadequate health service infrastructures and low health expenditure due to less developed economic status are thought to have resulted in inequity in neonatal and child health in the western region (2, 23). The most recent national survey on accessibility and availability of neonatal care resources showed that the density of health resources, the level of technical development, and educational background of healthcare professionals in western China still lagged behind those in other regions of China (20). Therefore, it is not surprising that the overall mortality of infants at <34 weeks in western China was almost three times that of eastern China, given that these infants were the most healthcare resource–consuming and high-level-skill requiring. Then, it is important to recognize the most efficient and economical way to improve the outcome of preterm infants cared for in less developed areas. Our results showed that the significant regional disparity of outcomes existed not only among the smallest infants, but also among those with relatively large gestational age. The overall mortality of infants at 32–33 weeks reached 10.2% in western China, more than four times that in eastern China. Given that infants at 32–33 weeks accounted for near half of infants at <34 weeks, the optimization of management for these infants may need to be prioritized in western China. Some evidence-based care practices that were not resource-intensive may also need to be better implemented in western China, such as the low antenatal steroids use rate we found in western China.

For eastern China, however, although with the lowest mortality, nearly one-fourth of infants at <34 weeks experienced death or any major morbidity during hospitalization. This rate of adverse composite outcome was significantly higher than that of developed countries (24, 25). Infants in eastern China did not show a decreased risk of individual morbidity compared with central and western China, either. Therefore, with more preterm infants who survived, hospitals in eastern China may need to pay more attention to reduce morbidities among survivors and to improving the intact survival of preterm infants. For infants with the youngest gestational age, no significantly improved outcome was observed in eastern China in our study, although this area possessed the most abundant neonatal care resources in China (20). Therefore, more investigations are needed targeting to improve the outcomes of the smallest infants. Our studies identified that several care practices in eastern China remained suboptimal compared with developed countries or even with other regions in China, such as inborn rate, antenatal steroids use, early CPAP, and breast milk feeding.

Infants in central China showed intermediate outcomes among the three regions. However, central China was associated with a significantly higher risk of severe brain injury, which required focused study to find out the reasons. Also, the lowest use rate of CPAP, longest duration of the first course of invasive ventilation, the lowest rate of breast milk feeding, latest initiation of feeds, and the longest duration of TPN in central China indicate that targeted quality improvement on ventilation and nutrition practices may be needed in this area.

DAMA is a critical cause related to mortality in China (22, 26). Our results identified that 17.7% of infants in western China were DAMA, the highest among all regions, contributing to the highest rate of overall mortality in western China. According to a survey, the cost of care was one of the most critical factors influencing the active management of extremely preterm infants in China (27). Lin et al. (28) showed that the mean total cost of infants survivors was 62,206 ± 39,762 CNY, which was nearly three times higher than the entire annual income of an urban resident in 2011 (19,118 CNY). Therefore, socioeconomic status may be one reason for the highest rate of DAMA in western China. Nevertheless, the DAMA rates were generally high across China. Overall, 6.3% of infants in eastern China were DAMA, with 18.2% DAMA rate among infants at <28 weeks. Therefore, intervention to guarantee more infants receive complete care is urgently needed in China and may contribute significantly to the overall mortality of preterm infants.

There are several limitations to our study. First, our study included only tertiary NICUs in large cities of three regions. Substantial variation exists between urban and rural areas within regions, so our results could not represent the situation in rural areas (1, 3, 20). Second, the study was hospital-based. Different rates of stillbirth or delivery room death would result in selection bias and underestimation of mortality. Third, the diagnosis of morbidities was not standardized in the study, which might result in misclassification of some morbidities.

## Conclusions

We identified marked disparities in outcomes and care practices of preterm infants born at <34 weeks' gestation in different regions of China. Infants in eastern China were at the lowest risk of mortality, but not major morbidities. Western China showed the highest mortality, even for infants born at 32–33 weeks. Further collaboration networking, benchmarking, and targeted quality improvement initiatives are needed to deal with region-specific problems, to reduce regional variations, and to improve overall outcomes of preterm infants in China.

## Data Availability Statement

The datasets presented in this article are not readily available because of other ongoing projects using the dataset. Requests to access the datasets should be directed to yuncao@fudan.edu.cn.

## Ethics Statement

The studies involving human participants were reviewed and approved by the Ethics Committee of the Children's Hospital of Fudan University [(2015) No. 28]. Written informed consent to participate in this study was provided by the participants' legal guardian/next of kin.

## Author Contributions

RB, SiJ, SL, ZL, and YC: study concept and design. SiJ, JG, and ShJ: acquisition, analysis, or interpretation of data. RB and SiJ: drafting of the manuscript and statistical analysis. RB, SiJ, JG, ShJ, SL, ZL, and YC: critical revision of the manuscript for important intellectual content. SL, ZL, and YC: study supervision. All authors contributed to the article and approved the submitted version.

## Funding

This study was funded by the China Medical Board (Grant Number 14-194 to YC) and the Canadian Institutes of Health Research (Grant Number CTP 87518 to SL). The funders had no role in the design and conduct of the study; collection, management, analysis, and interpretation of the data; preparation, review, or approval of the manuscript; and decision to submit the manuscript for publication.

## Conflict of Interest

The authors declare that the research was conducted in the absence of any commercial or financial relationships that could be construed as a potential conflict of interest.

## Publisher's Note

All claims expressed in this article are solely those of the authors and do not necessarily represent those of their affiliated organizations, or those of the publisher, the editors and the reviewers. Any product that may be evaluated in this article, or claim that may be made by its manufacturer, is not guaranteed or endorsed by the publisher.
