# Supplementary material for: Variation of Neonatal Outcomes and Care Practices for Preterm Infants <34 Weeks' Gestation in Different Regions of China: A Cohort Study
Source: Front Pediatr. 2021 Nov 11;9:760646. doi: 10.3389/fped.2021.760646 (PMC8636048; doi:10.3389/fped.2021.760646)
Supplement: Supplementary file 1 [file Data_Sheet_1.docx]

Supplementary Material

**Supplemental Table 1 The characteristics of three groups of hospitals**

|  | Eastern China | Central China | Western China |
| --- | --- | --- | --- |
|  | N=12 | N=9 | N=4 |
| **Type of hospitals** |  |  |  |
| Free-standing Children's **Hospital** | 5 | 3 | 1 |
| Perinatal center | 6 | 3 | 2 |
| General hospital | 1 | 3 | 1 |
| Teaching hospital | 12 | 5 | 2 |
| **Availability of healthcare** |  |  |  |
| Transport team | 8 | 6 | 3 |
| General Surgery | 7 | 9 | 4 |
| PDA Ligation | 8 | 9 | 3 |
| Cardiac Surgery | 7 | 7 | 3 |
| ECMO | 5 | 1 | 2 |
| MRI | 11 | 8 | 4 |

**Supplemental table 2**Comparison of unadjusted outcomes for preterm infants admitted to NICUs in three regions of China

|  | Gestation age | Eastern China | Central China | Western China | P |
| --- | --- | --- | --- | --- | --- |
| Mortality or any morbidity*, n/N (%) | ≤27^+6^ | 816/1188(68.7) | 298/419(71.1) | 198/263(75.3) | 0.095 |
|  | 28^+0^-31^+6^ | 1869/6593(28.4) | 1326/3725(35.6) | 1107/2489(44.5) | <0.001 |
|  | 32^+0^-33^+6^ | 629/6397(9.8) | 586/3925(14.9) | 618/2533(24.4) | <0.001 |
| Overall mortality, n/N (%) | ≤27^+6^ | 401/1188(33.8) | 188/419(44.9) | 150/263(57.0) | <0.001 |
|  | 28^+0^-31^+6^ | 520/6593(7.9) | 538/3725(14.4) | 614/2489(24.7) | <0.001 |
|  | 32^+0^-33^+6^ | 157/6397(2.5) | 183/3925(4.7) | 259/2533(10.2) | <0.001 |
| In-hospital mortality, n/N (%) | ≤27^+6^ | 211/973(21.7) | 98/314(31.2) | 61/163(37.4) | <0.001 |
|  | 28^+0^-31^+6^ | 214/6145(3.5) | 210/3239(6.5) | 208/1950(10.7) | <0.001 |
|  | 32^+0^-33^+6^ | 67/6169(1.1) | 54/3662(1.5) | 65/2235(2.9) | <0.001 |
| DAMA, n/N (%) | ≤27^+6^ | 215/1188(18.1) | 105/419(25.1) | 100/263(38.0) | <0.001 |
|  | 28^+0^-31^+6^ | 448/6593(6.8) | 486/3725(13.1) | 539/2489(21.7) | <0.001 |
|  | 32^+0^-33^+6^ | 228/6397(3.6) | 263/3925(6.7) | 298/2533(11.8) | <0.001 |
| Sepsis, n/N (%) | ≤27^+6^ | 146/1188(12.3) | 28/419(6.7) | 27/263(10.3) | <0.001 |
|  | 28^+0^-31^+6^ | 465/6593(7.1) | 202/3725(5.4) | 220/2489(8.8) | <0.001 |
|  | 32^+0^-33^+6^ | 150/6397(2.3) | 83/3925(2.1) | 116/2533(4.6) | <0.001 |
| Severe IVH or PVL, n/N (%) | ≤27^+6^ | 191/1002(19.1) | 71/304(23.4) | 40/181(22.1) | 0.216 |
|  | 28^+0^-31^+6^ | 397/6144(6.5) | 408/3315(12.3) | 136/2104(6.5) | <0.001 |
|  | 32^+0^-33^+6^ | 129/5655(2.3) | 217/3481(6.2) | 84/2306(3.6) | <0.001 |
| NEC, n/N (%) | ≤27^+6^ | 91/985(9.2) | 16/321(5.0) | 11/165(6.7) | 0.041 |
|  | 28^+0^-31^+6^ | 342/6308(5.4) | 121/3495(3.5) | 130/2142(6.1) | <0.001 |
|  | 32^+0^-33^+6^ | 109/6186(1.8) | 54/3801(1.4) | 86/2322(3.7) | <0.001 |
| BPD, n/N (%) | ≤27^+6^ | 622/1187(52.4) | 209/419(50.0) | 152/263(57.8) | 0.009 |
|  | 28^+0^-31^+6^ | 995/6593(15.1) | 739/3725(19.8) | 828/2489(33.3) | <0.001 |
|  | 32^+0^-33^+6^ | 255/6397(4.0) | 253/3925(6.5) | 416/2533(16.4) | <0.001 |
| Severe ROP, n/N (%) | ≤27^+6^ | 73/747(9.8) | 19/216(8.8) | 7/103(6.8) | 0.598 |
|  | 28^+0^-31^+6^ | 48/4590(1.1) | 44/2305(1.9) | 11/1425(0.8) | 0.002 |
|  | 32^+0^-33^+6^ | 6/2085(0.3) | 9/1572(0.6) | 0 | 0.045 |

* Mortality or any morbidity was defined as the occurance of overall death or any morbidity, including sepsis,necrotizing enterocolitis (NEC), severe intraventricular hemorrhage (IVH), periventricular leukomalacia (PVL), severe retinopathy of prematurity (ROP), and bronchopulmonary dysplasia (BPD).

**Supplemental table 3** Comparison of adjusted outcomes for preterm infants admitted to NICUs in three regions of China

|  | Gestation age | Eastern China | Model 1 |  |  | Model 2 |  |
| --- | --- | --- | --- | --- | --- | --- | --- |
|  |  |  | Central China | Western China |  | Central China | Western China |
| Mortality or any morbidity | ≤27^+6^ | Reference | 1.0 (0.6-1.9) | 1.7 (0.8-3.6) |  | 0.9 (0.5-1.7) | 1.7 (0.8-3.7) |
|  | 28^+0^-31^+6^ | Reference | 1.4 (0.8-2.3) | 2.4 (1.3-4.5) |  | 1.4 (0.9-2.3) | 2.4 (1.3-4.6) |
|  | 32^+0^-33^+6^ | Reference | 1.6 (0.9-2.8) | 2.8 (1.4-5.8) |  | 1.5 (0.9-2.5) | 3.1 (1.6-6.0) |
| Overall mortality | ≤27^+6^ | Reference | 1.5 (0.8-2.8) | 2.9 (1.3-6.6) |  | 1.2 (0.7-2.4) | 2.8 (1.2-6.4) |
|  | 28^+0^-31^+6^ | Reference | 2.0 (1.2-3.2) | 4.5 (2.4-8.4) |  | 1.9 (1.1-3.0) | 4.5 (2.4-8.4) |
|  | 32^+0^-33^+6^ | Reference | 1.9 (1.0-3.5) | 4.1 (1.9-8.9) |  | 1.7 (1.0-2.8) | 4.7 (2.5-8.9) |
| In-hospital mortality | ≤27^+6^ | Reference | 1.4 (0.7-2.7) | 3.0 (1.4-6.7) |  | 1.2 (0.6-2.2) | 3.1 (1.4-6.8) |
|  | 28^+0^-31^+6^ | Reference | 2.0 (1.3-3.3) | 4.5 (2.6-8.1) |  | 1.9 (1.2-3.1) | 4.5 (2.5-8.2) |
|  | 32^+0^-33^+6^ | Reference | 1.4 (0.7-2.9) | 3.0 (1.3-7.0) |  | 1.3 (0.7-2.3) | 3.5 (1.8-6.9) |
| DAMA | ≤27^+6^ | Reference | 1.5 (0.7-3.2) | 2.4 (0.9-6.3) |  | 1.4 (0.7-2.9) | 2.3 (0.9-5.9) |
|  | 28^+0^-31^+6^ | Reference | 2.0 (1.2-3.5) | 3.7 (1.9-7.3) |  | 1.9 (1.1-3.2) | 3.7 (1.9-7.2) |
|  | 32^+0^-33^+6^ | Reference | 1.9 (1.1-3.4) | 3.3 (1.6-6.6) |  | 1.7 (1.0-2.9) | 3.4 (1.7-6.6) |
| Sepsis | ≤27^+6^ | Reference | 0.5 (0.2-1.2) | 1.0 (0.3-2.9) |  | 0.5 (0.2-1.2) | 1.0 (0.3-3.0) |
|  | 28^+0^-31^+6^ | Reference | 0.8 (0.4-1.5) | 1.4 (0.6-3.3) |  | 0.8 (0.4-1.6) | 1.5 (0.6-3.3) |
|  | 32^+0^-33^+6^ | Reference | 1.0 (0.5-1.9) | 2.2 (1.0-5.0) |  | 1.0 (0.5-1.9) | 2.2 (1.0-5.0) |
| Severe IVH or PVL | ≤27^+6^ | Reference | 1.4 (0.7-2.8) | 2.3 (1.0-5.6) |  | 1.5 (0.7-3.4) | 2.8 (1.1-7.2) |
|  | 28^+0^-31^+6^ | Reference | 2.6 (1.2-5.8) | 2.0 (0.8-5.4) |  | 2.8 (1.2-6.4) | 2.1 (0.8-5.9) |
|  | 32^+0^-33^+6^ | Reference | 3.7 (1.1-12.4) | 2.0 (0.4-9.3) |  | 3.2 (0.9-11.5) | 2.3 (0.8-10.8) |
| NEC | ≤27^+6^ | Reference | 0.5 (0.2-1.2) | 0.7 (0.2-2.3) |  | 0.4 (0.2-1.2) | 0.7 (0.2-2.5) |
|  | 28^+0^-31^+6^ | Reference | 0.6 (0.3-1.1) | 1.0 (0.4-2.3) |  | 0.6 (0.3-1.2) | 0.9 (0.4-2.3) |
|  | 32^+0^-33^+6^ | Reference | 0.9 (0.4-1.9) | 1.6 (0.6-4.3) |  | 0.8 (0.4-1.7) | 1.9 (0.8-4.9) |
| BPD | ≤27^+6^ | Reference | 0.8 (0.3-1.9) | 1.2 (0.4-3.7) |  | 0.7 (0.3-1.7) | 1.3 (0.4-3.9) |
|  | 28^+0^-31^+6^ | Reference | 1.1 (0.5-2.5) | 2.1 (0.7-6.2) |  | 1.1 (0.5-2.5) | 2.2 (0.8-6.4) |
|  | 32^+0^-33^+6^ | Reference | N/A | N/A |  | N/A | N/A |
| Severe ROP | ≤27^+6^ | Reference | 1.0 (0.5-2.0) | 0.9 (0.3-2.7) |  | 0.8 (0.4-1.8) | 1.0 (0.4-2.9) |
|  | 28^+0^-31^+6^ | Reference | 2.1 (1.0-4.2) | 0.8 (0.3-2.2) |  | 2.1 (0.9-4.6) | 0.9 (0.3-2.6) |
|  | 32^+0^-33^+6^ | Reference | 3.3 (0.3-8.3) | N/A |  | 2.7 (0.3-7.9) | N/A |

Multilevel mixed-effects logistic regression models were used, accounting for the intracluster correlation among infants within individual sites.

Patient-level covariants adjusted in models.

Model 1: gender, gestational age, SGA, maternal hypertension, maternal diabetes.

Model 2: gender, gestational age, SGA, maternal hypertension, maternal diabetes, inborn, prenatal care, antenatal steriods,primigravida, caesearean section.
